# Cross-sectional differences in inhibitory control between adolescent badminton trainees and age-matched non-athletes: a Flanker task study

**DOI:** 10.3389/fpsyg.2026.1881972

**Published:** 2026-07-08

**Authors:** Xiaofeng Hu

**Affiliations:** School of Sports Training, Chengdu Sport University, Chengdu, Sichuan, China

**Keywords:** adolescence, badminton, conflict effect, executive function, Flanker task, inhibitory control, open-skill sport

## Abstract

**Background:**

Inhibitory control matures rapidly during early adolescence and supports academic achievement, emotion regulation, and social adjustment. Open-skill sports such as badminton, which combine aerobic demand, decision-making under perceptual uncertainty, and continuous response inhibition, are thought to confer greater cognitive benefits than closed-skill activities; direct behavioral evidence in amateur adolescent trainees aged 12–15 years nonetheless remains scarce.

**Methods:**

In a between-group cross-sectional design, 32 adolescents (mean age 13.5 years; 1–3 years of amateur badminton training, ≥3 sessions/week) and 30 age-matched, non-exercising controls completed an arrow-version Flanker task; trainees also completed a training-history questionnaire. Reaction time, error rate, and the Flanker conflict effect were compared with independent-samples *t*-tests, Cohen’s *d*, and Bayes factors, and corroborated by a mixed ANOVA, a trial-level mixed-effects model, and Inverse Efficiency Scores. Within the training group, Pearson correlations linked training years and weekly frequency to the four Flanker indices, with Benjamini–Hochberg correction across the eight tests.

**Results:**

The training group responded faster than controls under both congruent (*d* = 0.54) and incongruent (*d* = 1.07) conditions and made fewer errors under the incongruent condition (*d* = 1.02). The RT conflict effect (training *M* = 56.7 ms; control *M* = 94.3 ms; *d* = 1.41) and the error-rate conflict effect (*d* = 0.91) were both substantially smaller in the training group. Within the training group, training years correlated negatively with the RT conflict effect (*r* = −0.62, *p* < 0.001) and with incongruent-condition RT (*r* = −0.42, *p* = 0.016); weekly training frequency yielded correlations of the same direction but with lower statistical power.

**Conclusion:**

Adolescent badminton trainees aged 12–15 displayed substantially smaller Flanker conflict effects than age-matched non-exercising peers, and conflict-effect magnitude was negatively associated with training duration within the training group. Because the design is cross-sectional, causal inference is not warranted: badminton participation is associated with, rather than shown to cause, superior inhibitory control, and the differences should be read as an upper bound on any training effect. These findings are consistent with the open-skill exercise hypothesis and motivate longitudinal and randomized confirmation.

## Highlights

- Amateur badminton trainees aged 12–15 showed substantially smaller Flanker conflict effects than non-exercising peers.
- The effect was selective: large for the conflict effect, small for general processing speed.
- Training years correlated negatively with the RT conflict effect within the training group.
- A trial-level mixed-effects model and 2 × 2 mixed ANOVA both confirmed the group × condition interaction.
- An Inverse Efficiency Score analysis ruled out a speed–accuracy trade-off explanation.

## 1 Introduction

Inhibitory control, defined as the capacity to suppress prepotent responses and resist distracting information, is one of the three core components of executive function and develops rapidly during early adolescence (Diamond, 2013; Miyake et al., 2000). The 12–15-year range coincides with prefrontal-cortex maturation, including white-matter myelination, dorsolateral prefrontal pruning, and the integration of frontoparietal control networks. Behaviorally, these processes manifest as gains in interference suppression, response inhibition, and working-memory updating across the early-adolescent years. Executive-function trajectories during this window predict later academic performance, emotion regulation, and psychosocial adjustment, and environmental experiences that engage inhibitory-control circuits at this age can therefore shape long-term cognitive outcomes. Identifying activities that effectively recruit these circuits is therefore of practical relevance for school physical education, after-school programming, and family-level decisions about extracurricular activity.

### 1.1 Why the 12–15-year window matters

This developmental window warrants particular attention because of the trajectory of the circuits that the Flanker task indexes. Frontoparietal control regions, the dorsolateral prefrontal cortex, and the anterior cingulate cortex undergo protracted maturation during the second decade of life, with white-matter myelination, synaptic pruning, and functional connectivity refining well into the late teens. Pre-adolescent children, whose conflict-monitoring system is still distributed across less specialized regions, and young adults, whose system has approached relative stability, both differ from mid-adolescents in this respect. Mid-adolescents are in a transitional position: their inhibitory circuitry is consolidated enough to support adult-like task performance, yet still flexible enough that sustained experience may leave a measurable imprint on baseline efficiency.

This developmental position has two implications for the present study. First, environmental influences on inhibitory control in this window may produce larger behavioral differences than those reported in younger or older samples, a point revisited below when the present effect sizes are compared against meta-analytic benchmarks. Second, an explicit focus on amateur trainees rather than elite athletes is justified: amateur participation during this developmentally sensitive window is the realistic exposure profile for most adolescents, and is therefore the policy-relevant target.

### 1.2 Physical activity and inhibitory control in children and adolescents

A substantial literature shows that physical activity is associated with better inhibitory control in children and adolescents, with effects extending from acute exercise to long-term training (Best, 2010; Hillman et al., 2008; Verburgh et al., 2014). A meta-analysis of 23 randomized controlled trials (*N* = 2,857) reported that cognitively engaging physical-activity interventions produced reliable gains in executive function, with the largest effects observed for inhibitory control (SMD = 0.35); benefits were most pronounced when interventions exceeded 6 weeks, occurred more than twice weekly, and lasted at least 20 min per session (Mao et al., 2024). Within this literature, a distinction has been drawn between closed-skill activities (e.g., running, cycling), performed in stable and predictable environments, and open-skill activities (e.g., badminton, basketball, tennis), performed under continuously changing perceptual and tactical demands. Diamond and Ling proposed that the most effective cognitive interventions combine three ingredients (sustained aerobic load, complex decision-making, and rich social interaction), each of which open-skill sports satisfy (Diamond and Ling, 2016). Direct empirical support for the open-skill advantage in younger samples comes from a meta-analysis of 17 studies (*N* = 1,298) in children and adolescents aged 5–16, which reported significant intervention effects of open-skill exercise on inhibitory control, working memory, and cognitive flexibility, with optimal effects at moderate intensity and 3–5 sessions per week (Hu et al., 2024). Earlier behavioral work demonstrated the open- versus closed-skill dissociation on Flanker conflict-effect measures (Wang et al., 2013). Recent evidence specifically favors open-skill over closed-skill activity for inhibitory control in youth, from a large cross-sectional analysis of 9,898 children in the ABCD study (Shih et al., 2025) to controlled and randomized interventions (Aoyama, 2024; Gu et al., 2024) and recent meta-analytic and systematic reviews (Lai et al., 2024; Qiu et al., 2024).

Badminton is a prototypical open-skill sport. The shuttlecock typically reaches the receiver within a few hundred milliseconds, which compresses the available decision window. The opponent’s body orientation, racket angle, and arm motion convey partially deceptive cues that must be integrated, and overridden when misleading. Effective returns also require the simultaneous selection of one motor program and the inhibition of competing alternatives. These demands recruit interference suppression on virtually every rally, so badminton functions as a high-frequency trainer of inhibitory control. Behavioral and electrophysiological evidence converges on more efficient inhibitory control in badminton players. In a Flanker-task study, badminton athletes responded faster and with less variability than athletic controls and showed greater stability of midfrontal neural oscillations during conflict resolution, independent of aerobic fitness (Wang et al., 2017). In a change-signal task, badminton athletes exhibited reduced N2 and P3 amplitudes alongside shorter response-inhibition and re-engagement times, a pattern interpreted as efficient cortical processing combined with superior inhibitory control (Chen et al., 2019). More broadly, meta-analytic evidence shows that expert athletes outperform non-athletes on tasks indexing executive function and attentional control (Voss et al., 2010). Three gaps in this literature motivate the present study. First, almost all behavioral work has been conducted with elite adult athletes or university students, so the developmentally critical 12–15-year window remains largely unexamined. Second, most studies report only categorical between-group contrasts and offer little quantitative analysis of how training volume (years and weekly frequency) maps onto cognitive benefit. Third, the typical comparison contrasts elite athletes with non-athletes, which under-represents the much larger population of amateur adolescent trainees who are the actual target of school-based and recreational training programs.

### 1.3 Practical and policy context

The question also has practical relevance in the Chinese educational setting. National policy on the integration of sport and education (2020) calls for after-school sport programs to assume a larger share of adolescents’ daily activity time, while school physical-education curricula have been undergoing reform to expand both volume and skill diversity. Open-skill ball sports, in particular badminton, are widely offered as recreational options in middle-school and community settings. What has remained unclear is whether the typical amateur training intensity (three to five sessions per week of 1 h, sustained over 1–2 years) is associated with measurable cognitive benefits in adolescent participants, in addition to the general activity-level gains that any structured exercise would confer (Hu et al., 2024; Mao et al., 2024). The present study targets this exposure profile directly. If amateur badminton training during middle-school years is associated with better inhibitory control, this would supply concrete behavioral evidence to inform school physical-education curricula, after-school sport programming, and family-level decisions about extracurricular activity selection. These three translational endpoints are revisited in the Discussion.

### 1.4 The present study

The present study addressed these gaps with a cross-sectional comparison between adolescents engaged in amateur badminton training and age-matched non-exercising peers, using a standard arrow-version Flanker task (Eriksen and Eriksen, 1974). Three hypotheses were tested:

*H1*: The training group will respond faster than controls under the incongruent condition.

*H2*: The training group will show smaller RT and error-rate Flanker conflict effects than controls.

*H3*: Within the training group, training years and weekly training frequency will be negatively associated with incongruent-condition RT and with the conflict effect.

## 2 Materials and methods

### 2.1 Participants

Two groups of adolescents were recruited from Chengdu, China. The training group was drawn from three amateur badminton clubs and two middle-school badminton teams and met the following inclusion criteria: aged 12–15 years; systematic badminton training for 1–3 years; at least three weekly training sessions of ≥60 min; right-handed by self-report; and normal or corrected-to-normal vision. The control group was recruited from two middle schools in the same district and met the criteria of: aged 12–15 years; fewer than two weekly exercise sessions; no systematic training in any skill-based sport; right-handed; and normal or corrected-to-normal vision. Both groups satisfied common exclusion criteria: no head injury during the preceding 3 months; no diagnosis of attention-deficit/hyperactivity disorder, autism-spectrum disorder, or other neurodevelopmental conditions; no medication affecting the central nervous system; and no caffeine intake or vigorous exercise during the 24 h preceding testing. Across the training group, this corresponded to a weekly training volume of approximately 287 ± 68 min (session frequency × duration; full descriptive statistics in §3.1). Training records captured session frequency and duration but did not decompose exposure into technical drilling, tactical practice, and competitive match play; session-level activity logs are therefore recommended for future work (see Limitations).

Sixty-five adolescents were initially enrolled (33 in the training group and 32 in the control group). Three participants (one from the training group and two from the control group) were excluded prior to data analysis. Two prespecified criteria were applied: an error rate exceeding 50% under the incongruent condition, or an overall error rate exceeding three standard deviations above the group mean. Both criteria indicate non-compliance with task instructions or task performance below minimal proficiency. The final sample therefore comprised 32 training-group participants (17 male, 15 female) and 30 control-group participants (16 male, 14 female). The two groups did not differ significantly on age, BMI, height, weight, or sex distribution (Table 1).

**Table 1:** Demographic characteristics of the training and control groups (M ± SD).

Variable	Training (*n* = 32)	Control (*n* = 30)	Statistic	*p*	Cohen’s *d*
Age (years)	13.47 ± 1.08	13.57 ± 1.01	*t*(60) = −0.38	0.708	−0.10
Sex (M/F)	17/15	16/14	χ^2^(1) = 0.00	0.987	—
Height (cm)	158.49 ± 8.38	156.99 ± 9.00	*t*(60) = 0.68	0.500	0.17
Weight (kg)	48.38 ± 9.03	49.60 ± 10.09	*t*(60) = −0.50	0.617	−0.13
BMI (kg/m^2^)	19.13 ± 2.66	19.99 ± 3.07	*t*(60) = −1.18	0.242	−0.30

### 2.2 Apparatus and stimuli

**Figure 1:**
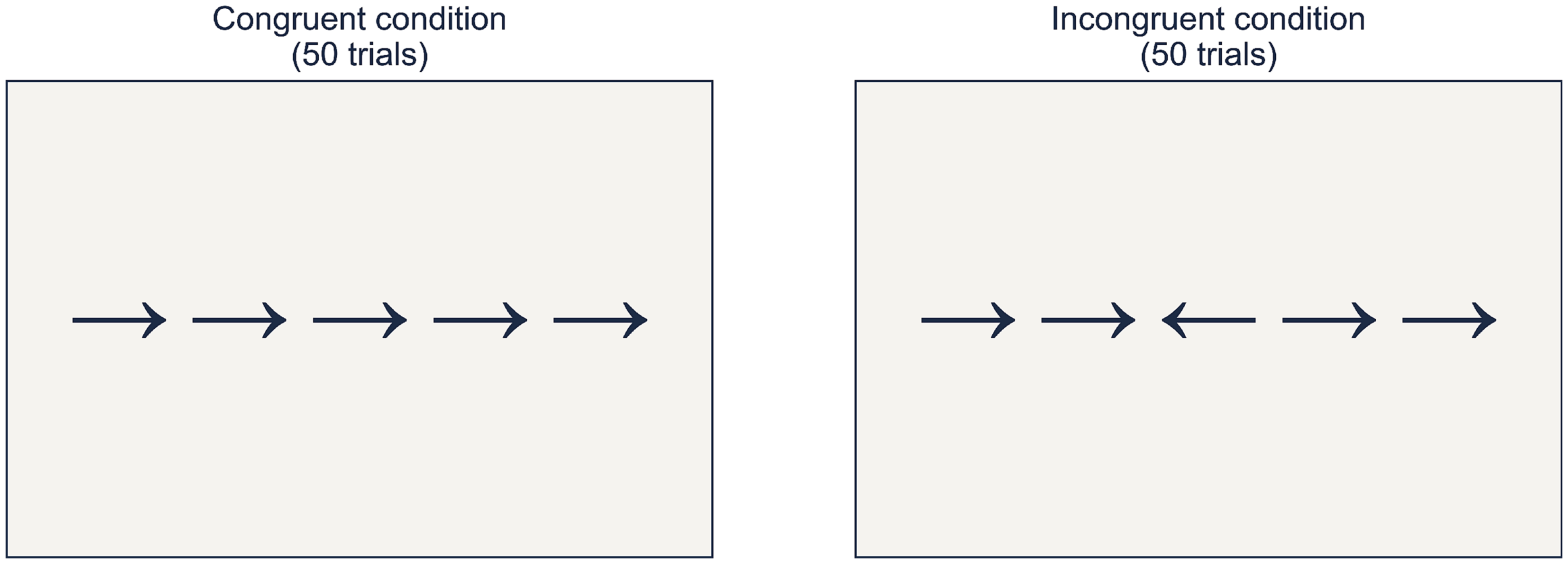
Stimulus examples for the arrow-version Flanker task. Top row: congruent array; bottom row: incongruent array.

Stimuli were presented on a 19-inch CRT monitor (1,024 × 768 resolution, 60 Hz refresh rate) viewed from approximately 60 cm in a quiet, dimly lit room at the Sport Psychology Laboratory of Chengdu Sport University. The arrow-version Flanker task was programmed in E-Prime 3.0 (Psychology Software Tools, Sharpsburg, PA, USA). Each stimulus array consisted of five horizontally arranged arrows (each subtending approximately 1.0° of visual angle; full array ≈ 5.2°) rendered in black on a light-grey background (RGB 200, 200, 200). Examples of congruent and incongruent arrays appear in Figure 1. The arrow version of the Flanker task is a widely used and well-validated behavioral index of interference control and inhibitory function (Eriksen and Eriksen, 1974; Diamond, 2013).

### 2.3 Flanker task

Each trial began with a central fixation cross (“+”) presented for 500 ms, followed by a 300-ms blank screen and then the arrow array, which remained on screen until the participant responded or 1,500 ms had elapsed. The inter-trial interval was jittered randomly between 500 and 800 ms. Participants responded with the right hand, pressing “F” with the index finger for a leftward-pointing center arrow and “J” with the middle finger for a rightward-pointing center arrow. A 16-trial practice block preceded the experimental block; participants had to reach ≥80% accuracy in practice to proceed. The experimental block consisted of 100 trials (50 congruent and 50 incongruent; left and right directions balanced; pseudo-random order), with a 30-s rest break after trial 50. Total task duration was approximately 8 min.

### 2.4 Procedure

Participants were tested individually in the laboratory described above. After parental written informed consent and adolescent written informed assent had been obtained, participants provided basic demographic information; training-group participants additionally completed a badminton-training questionnaire covering age at training onset, total training years, weekly training frequency, average session duration, and competition history. Participants then received standardized task instructions, completed the 16-trial practice block, and proceeded to the 100-trial experimental block once practice accuracy exceeded 80%. The study protocol was approved by the Institutional Review Board of Chengdu Sport University (approval no. CDSUIRB2025195; approval date 14 September 2025), and was conducted in accordance with the Declaration of Helsinki; written informed consent was provided by each participant’s legal guardian and written assent by each adolescent.

### 2.5 Data processing

Trials with reaction times below 150 ms or above 1,500 ms were excluded as outliers, as were trials exceeding ±3 *SD* of each participant’s mean. Across the 62 retained participants, 71 trials (1.15% of 6,200 total trials) were excluded under these criteria, all with RT < 150 ms (interpreted as anticipatory responses); no trial had RT > 1,500 ms, and none exceeded the ±3 *SD* threshold. Error-rate analyses used all retained trials, whereas mean RT analyses were restricted to correct trials. The Flanker conflict effect was operationalized as the difference between incongruent and congruent conditions, computed separately for RT and error rate; smaller values indicate stronger interference suppression.

Internal consistency. Split-half (odd–even, Spearman–Brown-corrected) reliabilities computed from the trial-level data were high for the underlying response times (congruent RT = 0.81; incongruent RT = 0.96) and lower for the conflict-effect difference score (0.38). This dissociation is expected: robust experimental effects with low between-subject variance yield highly reliable condition-level measurement but modest difference-score reliability, the so-called reliability paradox, documented for the Eriksen Flanker and related tasks by Hedge et al. (2018). Group-level inferences are unaffected; the modest difference-score reliability is, however, a caveat for the within-group dose–response correlation, which it likely attenuates (see Limitations).

### 2.6 Sample-size planning and screening cascade

Sample-size planning was anchored to prior open-skill versus closed-skill behavioral effects in the racket-sports literature. Wang and colleagues’ Flanker comparison of open- and closed-skill players reported an RT conflict-effect difference in the moderate-to-large range (Wang et al., 2013). Adopting Cohen’s *d* = 0.70 as a conservative target, an *a priori* analysis in G*Power 3.1 (independent-samples t-test, two-tailed, *α* = 0.05, 80% power) returned a required total N of approximately 68. Recruitment proceeded in two waves from three amateur badminton clubs and two middle-school badminton teams (training group) and two middle schools (control group) within the same district of Chengdu. Of 71 adolescents who initially expressed interest, 65 met inclusion criteria after telephone screening (no head injury, no neurodevelopmental diagnosis, no central-nervous-system medication; right-handed; weekly training and exercise frequencies meeting group-specific thresholds). All 65 were enrolled and tested; three were subsequently excluded for poor task compliance under the prespecified accuracy criteria reported above, leaving a final analyzed sample of *N* = 62. Handedness was assessed by a single self-report item rather than by the Edinburgh Handedness Inventory, a limitation revisited below.

### 2.7 Statistical analysis

All analyses were performed in Python 3.14 (NumPy, SciPy, pandas, statsmodels 0.14, pingouin 0.6; Vallat, 2018); equivalent SPSS / R workflows would yield numerically comparable results. Two-tailed *α* = 0.05 throughout, with effect sizes and 95% confidence intervals reported for every test in line with APA 7 reporting standards. These approaches are complementary rather than overlapping: the mixed ANOVA and the trial-level mixed-effects model test the group × condition interaction at the participant and single-trial levels, respectively; Bayes factors quantify the strength of evidence (including positive support for null effects); equivalence tests (TOST) evaluate whether the groups are demographically comparable; and Benjamini–Hochberg FDR correction controls multiplicity across the correlation tests.

#### 2.7.1 Normality and homogeneity of variance

Shapiro–Wilk *W* tests examined deviations from normality for each dependent variable in each group. Levene’s *F* (median-centered, more robust under non-normality) tested homogeneity of variance between groups. When Levene indicated violation (*p* < 0.05), Welch’s correction was applied to the corresponding independent-samples *t-*test in place of the Student version. Trial exclusion proceeded under the *a priori* rules described in §2.5 (71 trials, 1.15% of 6,200, all anticipatory responses with RT < 150 ms).

#### 2.7.2 Effect sizes and confidence intervals

For independent-samples comparisons, Cohen’s d was computed from the pooled standard deviation (Cohen, 1988), and its 95% confidence interval was derived from the analytical Hedges standard-error formula. For Pearson correlations, the 95% confidence interval was computed via Fisher’s z transformation. For mixed-design ANOVA, partial *η^2^* was reported.

#### 2.7.3 Equivalence testing for demographic null comparisons

Because the goal of demographic balance checks is to support rather than to reject the equivalence of groups, two one-sided tests (TOST) were additionally performed on each demographic variable with the smallest effect size of interest (SESOI) set at Cohen’s *d* = 0.5. A TOST *p* < 0.05 supports the claim of statistical equivalence within ±0.5 SD; a non-significant TOST result indicates that the available sample is underpowered to demonstrate equivalence at this threshold and that conventional null t tests should be interpreted accordingly.

#### 2.7.4 Group × condition interaction

A 2 (group: training vs. control) × 2 (condition: congruent vs. incongruent) mixed ANOVA tested the group × condition interaction directly. A significant interaction would indicate that the conflict effect (incongruent minus congruent) differs reliably between groups, the central test of the open-skill exercise hypothesis as instantiated in this study.

#### 2.7.5 Trial-level linear mixed-effects model

To make full use of the trial-level structure (≈100 trials per participant) rather than reducing the data to participant-level means, a linear mixed-effects model was additionally fitted on RT from correct, retained trials (formula RT ∼ condition × group), with by-participant random intercepts plus random slopes for condition (REML estimation; statsmodels.formula.api.mixedlm). Including a random slope for condition, in addition to a random intercept, is recommended for confirmatory tests of within-subjects manipulations to avoid inflated Type I error rates (Barr et al., 2013). The condition × group fixed-effect coefficient provides the trial-level estimate of the conflict-effect difference between groups; its precision is substantially greater than that of the participant-level t-test counterpart.

#### 2.7.6 Bayesian evidence (Bayes factor)

For each primary group comparison and for each within-training correlation, the Bayes factor *BF*_10_ (the evidence ratio for the alternative over the null) was computed using the JZS prior implementation in pingouin. By convention, *BF*_10_ > 3 indicates moderate evidence for the alternative; *BF*_10_ > 10 strong; *BF*_10_ > 30 very strong; *BF*_10_ > 100 extreme. Conversely, *BF*_10_ < 1/3 supports the null. Bayes factors were adopted because, unlike null-hypothesis significance tests, they quantify the strength of evidence and can provide positive support for a null effect, which is essential for interpreting the small, non-significant general-speed difference.

#### 2.7.7 Speed–accuracy trade-off via the inverse efficiency score

Because RT and error-rate effects could in principle reflect a speed–accuracy trade-off rather than genuinely more efficient inhibitory control, the present study computed the Inverse Efficiency Score (IES = RT / [1 − error-rate]) per participant per condition (Bruyer and Brysbaert, 2011) and the corresponding IES conflict effect. A group-difference pattern on IES that aligns in direction and magnitude with the RT pattern rules out a speed–accuracy trade-off explanation.

#### 2.7.8 Within-training correlations and multiple-comparison correction

Pearson correlations were computed between the two training-volume indices (training years; weekly training frequency) and each of the four Flanker indices (congruent-condition RT, incongruent-condition RT, RT conflict effect, error-rate conflict effect), yielding eight tests. *p* values were adjusted using the Benjamini–Hochberg false-discovery-rate procedure (Benjamini and Hochberg, 1995). The Benjamini–Hochberg procedure was preferred over Bonferroni correction because it controls the false-discovery rate while preserving greater statistical power across the eight correlated tests.

#### 2.7.9 Sensitivity analyses for the primary correlation

For the primary within-training correlation (training years × RT conflict effect), influence diagnostics (Cook’s distance, leverage, and externally studentized residuals) were inspected to identify potentially influential participants. The correlation was refit excluding flagged observations as a sensitivity check.

#### 2.7.10 Power

A sensitivity analysis using G*Power 3.1 (Faul et al., 2007) indicated that the present sample (*n*_training_ = 32, *n*_control_ = 30) provided ≥80% power (*α* = 0.05, two-tailed) to detect between-group differences of Cohen’s *d* ≥ 0.72 and within-group Pearson correlations of |*r*| ≥ 0.48.

## 3 Results

### 3.1 Demographic equivalence of the two groups

The two groups did not differ significantly on age, sex distribution, height, weight, or BMI (Table 1). For each of the four continuous demographic variables, Cohen’s *d* effect sizes fell within the small range (|*d*| ≤ 0.30), and the 95% confidence intervals around each d spanned zero. Two one-sided tests (TOST) with a SESOI of *d* = 0.5 returned *p* values in the range 0.058–0.217 across the four continuous variables; none reached the conventional *α* = 0.05 threshold for a formal equivalence claim. It is therefore concluded only that the two groups did not differ at *α* = 0.05 under the standard null framing, and that the present sample size is underpowered to support a positive equivalence claim within ±0.5 *SD*. Within the training group, participants reported on average 2.12 ± 0.68 years of badminton training, 3.81 ± 0.82 sessions per week, and 75.6 ± 12.4 min per session.

### 3.2 Reaction times in the flanker task

**Figure 2:**
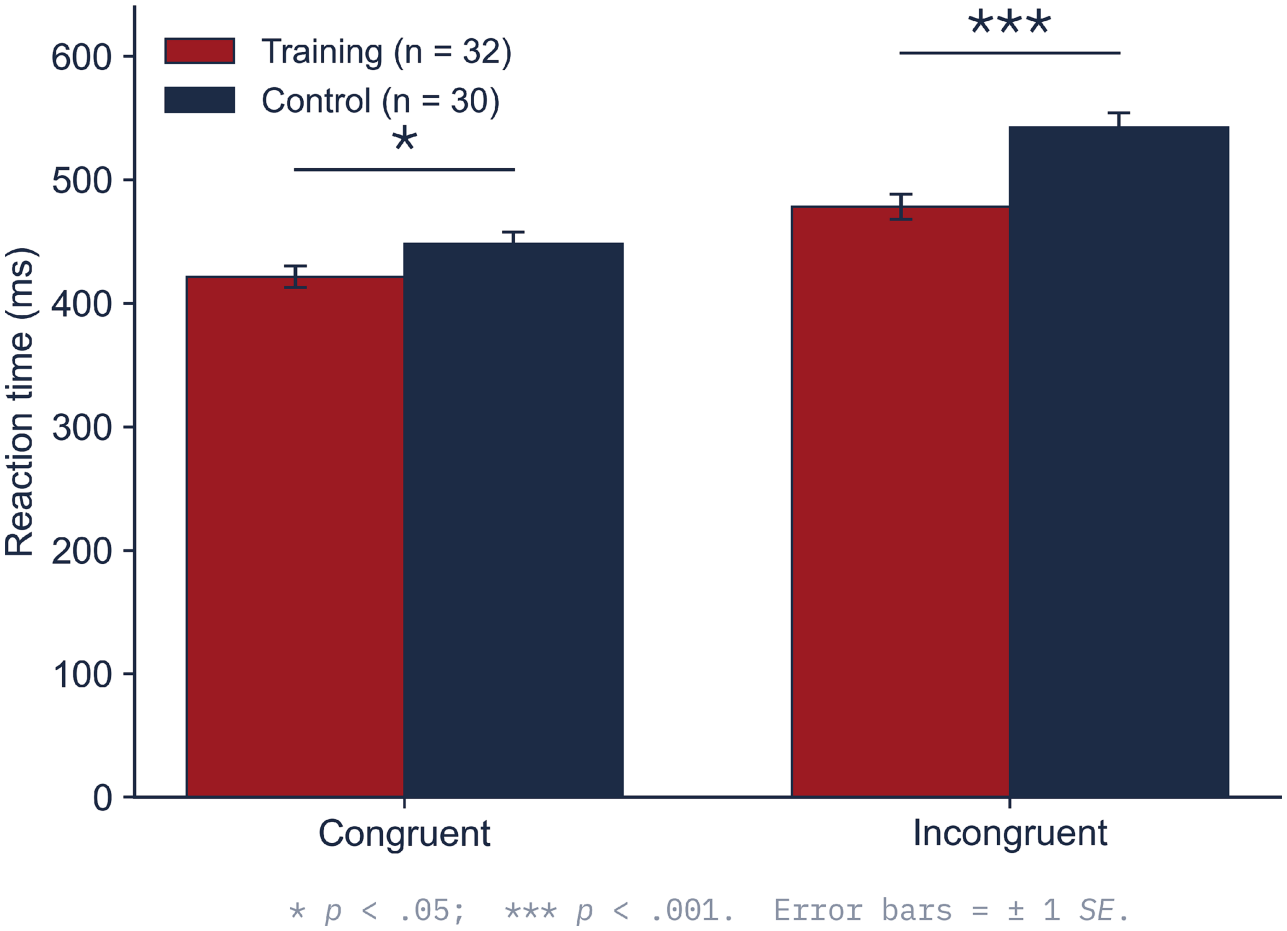
Reaction times (ms) by condition and group. Error bars represent ±1 SE.

Group differences in RT under the two conditions are summarized in Table 2 and Figure 2. Under the congruent condition, the training group (*M* = 421.5, *SD* = 48.3 ms) responded significantly faster than controls (*M* = 448.3, *SD* = 51.2 ms), *t*(60) = −2.12, *p* = 0.038, *d* = −0.54, 95% CI [−1.05, −0.03], *BF*_10_ = 1.7. Under the incongruent condition, the training-group advantage was more pronounced (training: *M* = 478.2, *SD* = 56.7 ms; controls: *M* = 542.6, *SD* = 63.8 ms), *t*(60) = −4.21, *p* < 0.001, *d* = −1.07, 95% CI [−1.60, −0.54], BF_10_ = 2.5 × 10^2^, very strong Bayesian evidence for a group difference. Levene’s *F* did not indicate a violation of homogeneity for either RT comparison, so Student’s t was used.

**Table 2:** Reaction time on the Flanker task (M ± SD, in ms).

Condition	Training (*n* = 32)	Control (*n* = 30)	Statistic	*p*	Cohen’s *d*
Congruent	421.50 ± 48.30	448.30 ± 51.20	*t*(60) = −2.12	0.038	−0.54
Incongruent	478.20 ± 56.69	542.60 ± 63.81	*t*(60) = −4.21	< 0.001	−1.07

Values are M ± SD. Group comparisons are independent-samples *t*-tests (two-tailed); Welch’s *t* with adjusted degrees of freedom is reported where Levene’s test indicated unequal variances. Cohen’s d is based on the pooled SD. Exact *p*-values are reported; *n* = 32 (training), 30 (control).

### 3.3 Error rates

**Figure 3:**
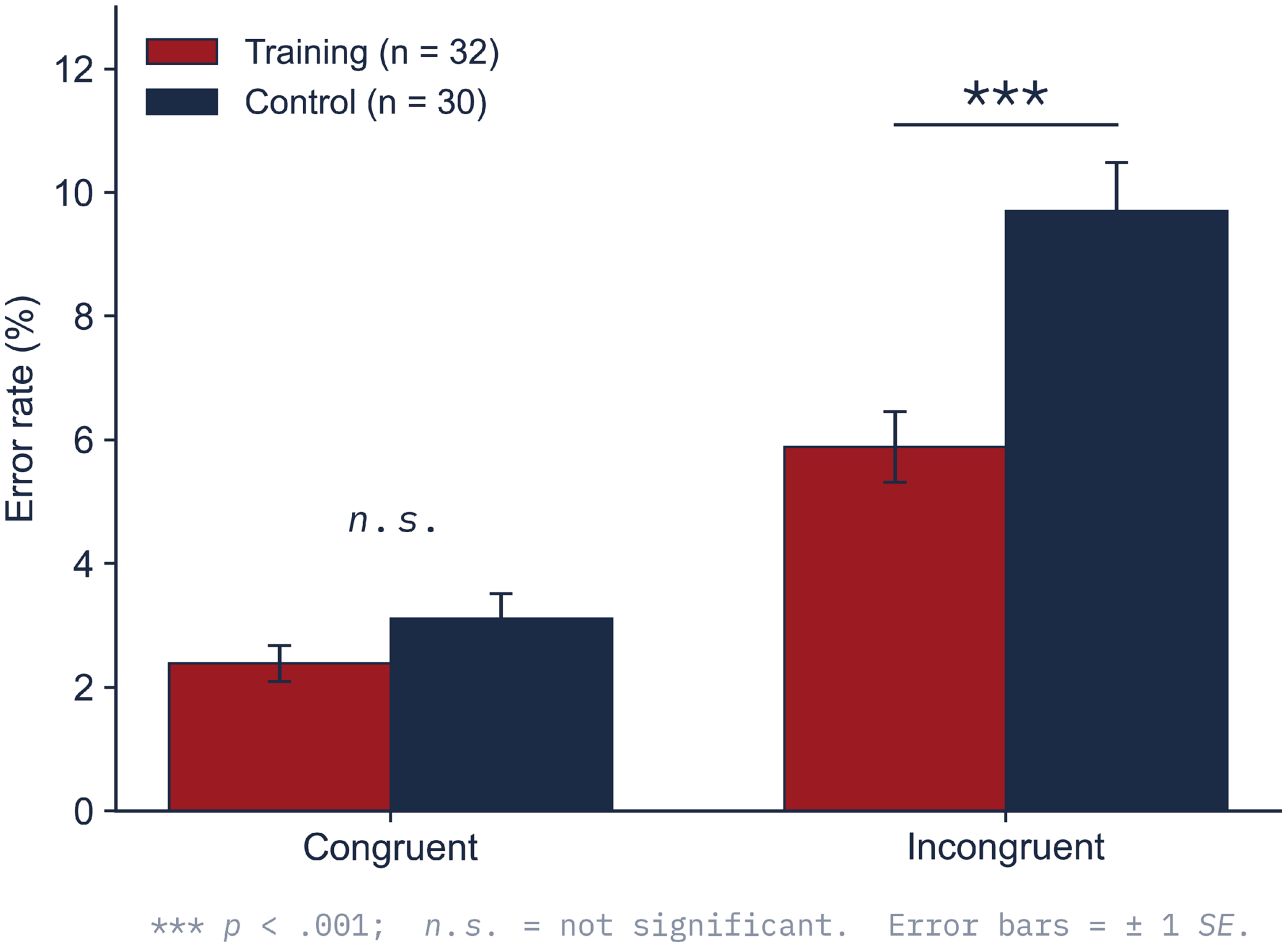
Error rates (%) by condition and group. Error bars represent ±1 SE.

Error rates are summarized in Table 3 and Figure 3. The between-group difference under the congruent condition was non-significant (training: *M* = 2.4 ± 1.6%; controls: *M* = 3.1 ± 2.2%; *t*(60) = −1.49, *p* = 0.142, *d* = −0.38, 95% CI [−0.88, +0.12], *BF*_10_ = 0.66). Bayesian evidence was inconclusive (*BF*_10_ between 1/3 and 3). Under the incongruent condition, Levene’s *F* indicated a homogeneity violation, so Welch’s correction was applied; the training group made significantly fewer errors than controls (training: *M* = 5.9 ± 3.2%; controls: *M* = 9.7 ± 4.3%; *t*(53.6) = −3.93, *p* < 0.001, *d* = −1.01, 95% CI [−1.54, −0.48], *BF*_10_ = 1.1 × 10^2^). The parallel pattern across RT and error rate rules out a speed–accuracy trade-off explanation, a conclusion formalized in §3.8 below.

**Table 3:** Error rate on the Flanker task (M ± SD, in %).

Condition	Training (*n* = 32)	Control (*n* = 30)	Statistic	*p*	Cohen’s *d*
Congruent	2.38 ± 1.64	3.11 ± 2.18	*t*(60) = −1.49	0.142	−0.38
Incongruent	5.88 ± 3.22	9.70 ± 4.30	*t*(53.6) = −3.93	< 0.001	−1.01

### 3.4 Flanker conflict effects

RT conflict effects (incongruent minus congruent) were significantly smaller in the training group (*M* = 56.7, *SD* = 22.4 ms) than in controls (*M* = 94.3, *SD* = 30.1 ms), *t*(60) = −5.61, *p* < 0.001, *d* = −1.42, 95% CI [−1.98, −0.87], *BF*_10_ = 2.3 × 10^4^, extreme Bayesian evidence for a group difference. The error-rate conflict effect followed the same pattern, with Welch’s correction applied due to a Levene violation (training: *M* = 3.5 ± 2.7%; controls: *M* = 6.6 ± 3.9%; *t*(50.8) = −3.62, *p* < 0.001, *d* = −0.93, 95% CI [−1.45, −0.41], *BF*_10_ = 47). Both effects exceeded Cohen’s large-effect benchmark (*d* ≥ 0.80) (Cohen, 1988) (Table 4).

**Table 4:** Flanker conflict effects in the two groups.

Conflict-effect index	Training (*n* = 32)	Control (*n* = 30)	Statistic	*p*	Cohen’s *d*
RT conflict effect (ms)	56.70 ± 22.43	94.29 ± 30.06	*t*(60) = −5.61	< 0.001	−1.42
Error conflict effect (%)	3.50 ± 2.67	6.59 ± 3.90	*t*(50.8) = −3.62	< 0.001	−0.93

### 3.5 Dose–response relationships within the training group

**Figure 4:**
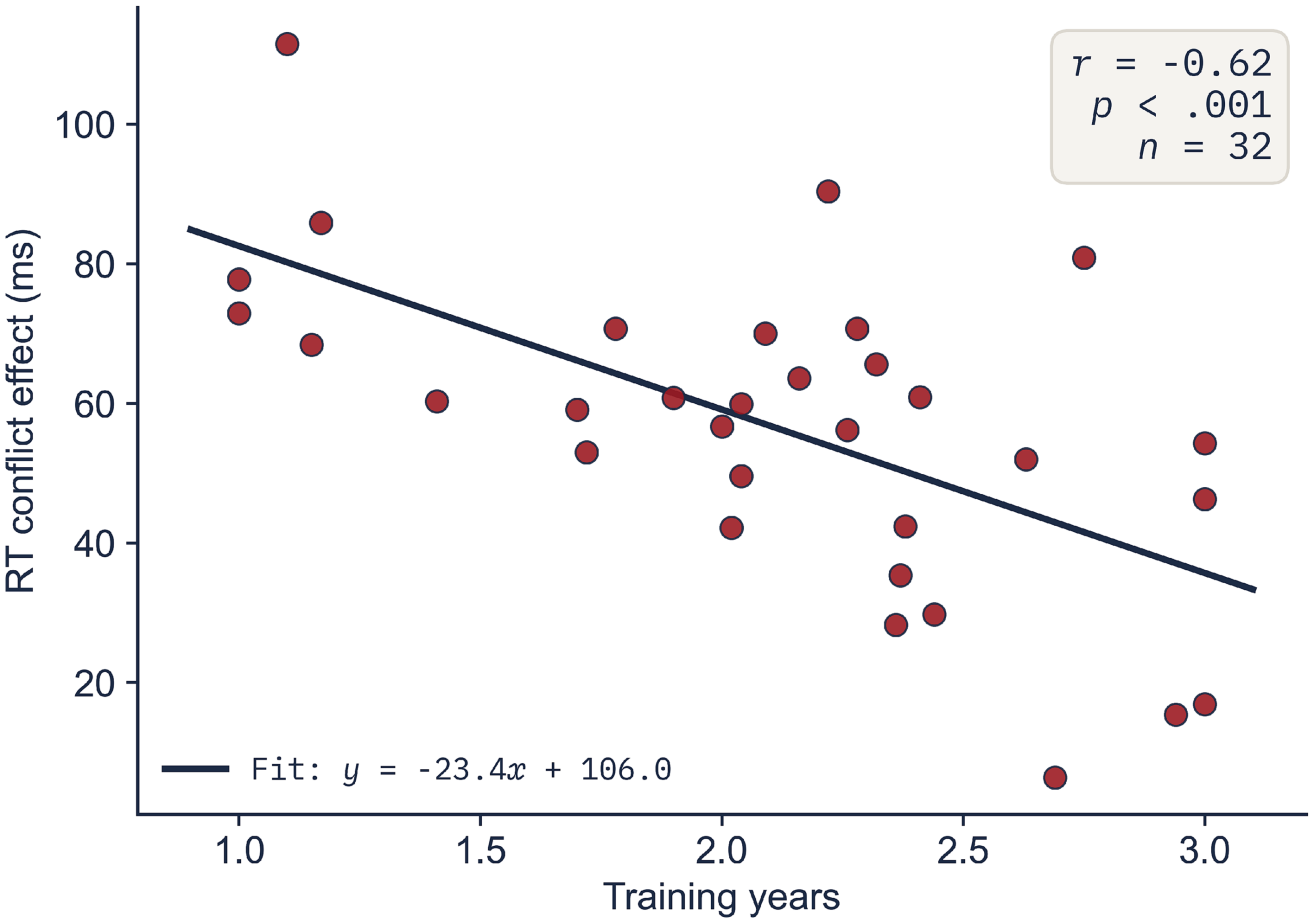
Scatterplot of training years against the RT conflict effect within the training group (*n* = 32). Solid line shows the linear least-squares fit; *r* = −0.62, *p* < 0.001.

Pearson correlations between training-volume indices and Flanker performance within the training group (*n* = 32) are presented in Table 5 and Figure 4. Training years correlated significantly with the RT conflict effect (*r* = −0.62, 95% CI [−0.80, −0.34], *p* < 0.001, *p*-FDR = 0.001, *BF*_10_ = 2 × 10^2^) and with incongruent-condition RT (*r* = −0.42, 95% CI [−0.67, −0.09], *p* = 0.016, *p*-FDR = 0.041, *BF*_10_ = 3.6); both survived Benjamini–Hochberg FDR correction across the eight tests. The correlation with congruent-condition RT was non-significant (*r* = −0.21, *p* = 0.246, *BF*_10_ = 0.42), and the correlation with the error-rate conflict effect was significant before but not after FDR correction (*r* = −0.37, *p* = 0.036, *p*-FDR = 0.057, BF_10_ = 1.8). Weekly training frequency showed a parallel pattern: the RT conflict effect (*r* = −0.52, *p* = 0.002, *p*-FDR = 0.009, *BF*_10_ = 20) and incongruent-condition RT (*r* = −0.40, *p* = 0.025, *p*-FDR = 0.050, *BF*_10_ = 2.5) both reached FDR-corrected significance, with congruent RT and the error-rate conflict effect remaining non-significant.

**Table 5:** Pearson correlations between training-volume indices and Flanker performance within the training group (*n* = 32).

Training-volume index	Congruent RT	Incongruent RT	RT conflict effect	Error conflict effect
Training years	−0.21	−0.42*	−0.62**	−0.37
Weekly training frequency	−0.22	−0.40	−0.52**	−0.26

Values are Pearson *r* within the training group (*n* = 32). Significance is based on Benjamini–Hochberg FDR-corrected p-values across the eight tests: *p-FDR < 0.05, **p-FDR <0.01. The RT conflict effect survived FDR correction for both indices (training years *p*-FDR = 0.001; weekly frequency *p*-FDR = 0.009), as did incongruent-condition RT (*p*-FDR = 0.041 and 0.050); congruent-condition RT and the error-rate conflict effect did not.

Sensitivity to influential observations. Influence diagnostics on the primary training-years × RT-conflict relationship identified five participants exceeding conventional Cook’s distance (>4/n) or leverage (>2 k/n) thresholds (T04, T05, T06, T18, T26). Refitting the correlation excluding these observations yielded *r* = −0.59, *p* = 0.001, *n* = 27, virtually identical to the full-sample estimate, indicating that the dose–response signal does not depend on any small subset of participants.

### 3.6 Group × condition interaction (2 × 2 mixed ANOVA)

**Figure 5:**
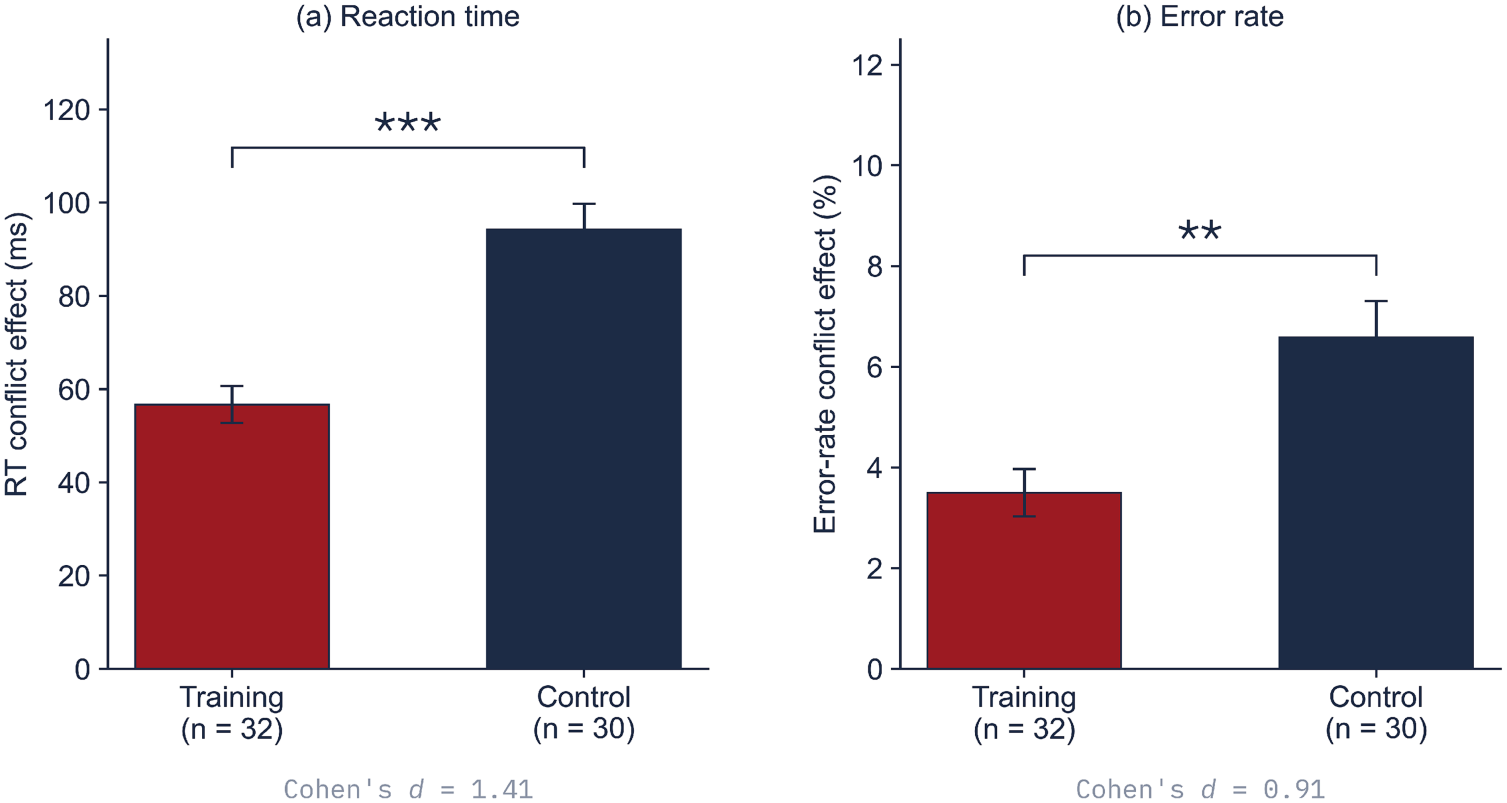
Flanker conflict effect by group. **(a)** Reaction-time conflict effect; **(b)** error-rate conflict effect. Bars show group means; error bars represent ±1 SE. ^**^p < 0.01; ^***^p < 0.001.

A 2 (group) × 2 (condition) mixed ANOVA on reaction time confirmed a significant main effect of group, *F*(1, 60) = 11.20, *p* = 0.001, partial *η^2^* = 0.16; a very large main effect of condition (congruent vs. incongruent), *F*(1, 60) = 499.18, *p* < 0.001, partial *η^2^* = 0.89; and, critically, a significant group × condition interaction, *F*(1, 60) = 31.43, *p* < 0.001, partial *η^2^* = 0.34. The same pattern held for error rate: significant effects of group, *F*(1, 60) = 12.80, *p* < 0.001, partial *η^2^* = 0.18; condition, *F*(1, 60) = 140.27, *p* < 0.001, partial *η^2^* = 0.70; and group × condition interaction, *F*(1, 60) = 13.41, *p* = 0.001, partial *η^2^* = 0.18. The interaction terms formalize, within a single inferential test, the central finding that the conflict-effect magnitude is reliably smaller in the training group than in controls (Figure 5).

### 3.7 Trial-level linear mixed-effects model

To exploit the full trial-level structure (5,815 retained correct trials across 62 participants) and to provide a precision-weighted estimate of the group × condition interaction, a linear mixed-effects model was fitted on single-trial RT with fixed effects of condition (0 = congruent, 1 = incongruent), group (0 = control, 1 = training), and their interaction, and by-participant random intercepts plus random slopes for condition. The estimated coefficients (in milliseconds) were: intercept = 448.6 (the control-group mean RT under the congruent condition); condition = +92.8 (the conflict-effect magnitude under the control-group baseline, *z* = 18.20, *p* < 0.001); group = −26.6 (the training-group RT advantage under the congruent condition, *z* = −2.09, *p* = 0.036); and group × condition = −36.0 (the additional reduction in the conflict effect attributable to training-group membership, *z* = −5.11, *p* < 0.001, 95% CI [−49.9, −22.2]). The trial-level interaction estimate is substantively identical to the participant-level *Δ* in conflict effect (94.3–56.7 = 37.6 ms) but rests on more than 5,800 trials and is therefore considerably more precise.

### 3.8 Speed–accuracy trade-off ruled out via inverse efficiency score

If the training-group advantage on incongruent trials reflected a bias toward faster but less accurate responding, a speed–accuracy trade-off explanation would be supported. To formally adjudicate, the present study computed the Inverse Efficiency Score (IES = RT / [1 − error-rate]) per participant per condition, and submitted the IES conflict effect to the same between-groups *t-*test as RT and error rate. The training group exhibited substantially smaller IES under the incongruent condition (training *M* = 509.5, *SD* = 69.3; control *M* = 603.5, *SD* = 85.5; *t*(60) = −4.74, *p* < 0.001, *d* = −1.21, 95% CI [−1.75, −0.67]) and a substantially smaller IES conflict effect (training *M* = 77.3, *SD* = 32.0 ms; control *M* = 140.3, *SD* = 52.3 ms; *t*(60) = −5.67, *p* < 0.001, *d* = −1.46, 95% CI [−2.02, −0.90]). The direction and magnitude of the IES effects mirror those of the RT effects, formally ruling out a speed–accuracy trade-off as an alternative explanation for the present pattern.

## 4 Discussion

### 4.1 Summary of main findings

This cross-sectional study compared inhibitory control between adolescents engaged in amateur badminton training and age-matched non-exercising peers. Three main findings emerged. First, the training group responded faster than controls under both conditions, with a substantially larger advantage under the incongruent condition (H1 supported). Second, both the RT and error-rate Flanker conflict effects were significantly smaller in the training group, with very large effect sizes (H2 supported). Third, within the training group, training years and weekly training frequency were negatively associated with incongruent-condition RT and with the RT conflict effect, with training years yielding the most robust dose–response signal (H3 partially supported; see qualifications below). Two of these results constitute the primary contributions of the study and are foregrounded in the sections that follow: the selectivity of the badminton advantage (large for the conflict effect, *d* = 1.41, but small for general processing speed, *d* = 0.54) and the within-group dose–response gradient (training years × RT conflict effect, *r* = −0.62). The general reaction-time difference, although reliable, is the less diagnostic finding.

### 4.2 Open-skill sport and inhibitory control in adolescence

The disproportionately large effect of badminton training on the Flanker conflict effect (*d* = 1.41) relative to general processing speed under the congruent condition (*d* = 0.54) is consistent with the open-skill exercise hypothesis (Diamond and Ling, 2016; Hu et al., 2024). Diamond and Ling argued that activities combining sustained aerobic load with complex decision-making and rich social interaction should selectively enhance executive-function components such as inhibitory control, beyond any generic improvement in perceptual or motor speed (Diamond and Ling, 2016). Badminton satisfies all three ingredients: rallies impose aerobic demand; opponent deception and rapid shuttlecock trajectories require continuous decision-making under uncertainty; and the training environment involves sustained peer and coach interaction. The conflict-effect reduction far exceeded general RT reduction. This dissociation provides converging behavioral evidence that the cognitive benefit of badminton training does not reflect a uniformly faster perceptual–motor system, but a selectively more efficient interference-control system. This pattern is consistent with prior reports of greater midfrontal oscillatory stability during conflict resolution in badminton athletes (Wang et al., 2017) and of reduced N2/P3 amplitudes during response inhibition (Chen et al., 2019).

#### 4.2.1 Convergence with the badminton neuroimaging literature

Although the present data are purely behavioral, two converging lines of neural evidence in badminton populations help to anchor the present pattern within a plausible neurobiological framework. The first concerns midfrontal oscillatory dynamics during conflict resolution. Wang and colleagues (Wang et al., 2017) reported that motor expertise in racket sports modulated the temporal stability of midfrontal theta-band oscillations during a conflict task, with badminton-trained participants showing more stable conflict-related midfrontal signatures than athletic controls. Critically, that effect held after controlling for aerobic fitness, suggesting that the modulation reflected something specific to open-skill training rather than a generic cardiovascular pathway. The second line concerns event-related potentials during response inhibition. In a change-signal task, Chen and colleagues (Chen et al., 2019) observed reduced N2 and P3 amplitudes alongside shorter re-engagement times in badminton athletes, a pattern interpreted as more efficient cortical processing rather than diminished inhibitory engagement *per se*.

Two features of the present behavioral pattern dovetail with these neural findings. First, the selective enlargement of the training-group advantage on the incongruent compared with the congruent condition mirrors the conflict-specific neural signatures reported in Wang and colleagues. Second, the parallel improvement in speed and accuracy under conflict, that is, the absence of a speed–accuracy trade-off, is consistent with the more efficient cortical processing inferred from Chen and colleagues’ ERP work, rather than with a simple bias toward faster but more error-prone responding. These are, however, circumstantial alignments. The present study collected no neural data, and the inference of a midfrontal conflict-monitoring mechanism (Botvinick et al., 2001) is therefore advanced only as a theoretical anchor. Mapped onto candidate substrates, the conflict-specific enlargement of the training-group advantage (*d* = 1.41 for the conflict effect versus *d* = 0.54 for general speed) is consistent with more efficient engagement of the anterior-cingulate conflict-monitoring and dorsolateral-prefrontal control system implicated in Flanker interference resolution (Botvinick et al., 2001), whereas the within-group dose–response gradient (*r* = −0.62) is consistent with experience-dependent tuning of this fronto-cingulate network. These mappings are interpretive rather than evidential, since no neural data were collected here.

An additional consideration that the neural literature raises but the present design cannot adjudicate is whether badminton training acts on the same conflict-monitoring substrate as closed-skill aerobic training, only with a stronger effect, or whether it engages partially distinct circuits, for instance, those subserving anticipatory motor planning and stimulus–response remapping under uncertainty. Future work pairing the behavioral design used here with EEG or fMRI in the same adolescent age range would help disambiguate these possibilities; absent such data, the present findings should be read as consistent with, but not diagnostic of, any specific neural model.

#### 4.2.2 Speed–accuracy and dual-process considerations

An additional formal test strengthens this interpretation. Inverse Efficiency Scores, which collapse speed and accuracy into a single metric and therefore detect any cross-domain trade-off, showed the same group × condition pattern as RT and error rate (§3.8), with the IES conflict-effect difference exceeding Cohen’s large-effect benchmark (*d* = −1.46). A purely speed–accuracy-trade-off account would have predicted attenuation or reversal of the group difference on IES; the observed amplification rules out this account. Had trained adolescents simply been biased toward faster responding, their incongruent error rate should have equalled or exceeded that of controls; instead, both speed and accuracy improved in parallel. From a dual-process perspective, the present pattern is more compatible with selective enhancement of top-down conflict resolution than with general gains in automatic, stimulus-driven processing. A purely automatic-route account would predict comparable training-group advantages under congruent and incongruent conditions and across speed and accuracy dimensions; the asymmetric pattern observed here instead points to selective upgrading of the controlled, effortful component of inhibition. These behavioral data are not interpreted as direct evidence for any specific neural mechanism. The prefrontal–anterior-cingulate conflict-monitoring circuit (Botvinick et al., 2001) offers a plausible theoretical anchor for the present effects, but mechanistic claims would require neuroimaging or electrophysiological data, neither of which were collected in the present study.

### 4.3 Dose–response patterns

The within-group correlation between training years and the RT conflict effect (*r* = −0.62; see Section 3.5) provides the clearest dose–response signal in the present dataset, consistent with the cumulative neuroplasticity hypothesis (Hillman et al., 2008). Hillman and colleagues framed cumulative aerobic training as a multi-pathway influence on brain structure and function, including elevations in brain-derived neurotrophic factor (BDNF), increases in hippocampal volume, and gains in prefrontal cerebral blood flow (Hillman et al., 2008). For an open-skill sport such as badminton, an additional pathway (repeated practice of interference control in ecologically rich environments) may contribute to the dose–response gradient observed here. These mechanisms remain, however, speculative in the absence of neural data. Because the conflict-effect difference score is of modest reliability (the reliability paradox; see Section 2), this dose–response correlation is likely attenuated, so *r* = −0.62 is best read as a conservative lower bound.

Within the amateur training range sampled (1–3 years; ≥3 sessions/week), training years predicted conflict-effect magnitude more reliably than weekly frequency did. Frequency correlations were underpowered at the present sample size (achieved power = 0.21–0.65), and strong claims about a weekly-frequency dose–response are therefore avoided. A further dissociation within the correlation pattern warrants brief comment: training years did not significantly predict congruent-condition RT (*r* = −0.21, n.s.) but did predict the conflict effect (*r* = −0.62). This dissociation is consistent with the selective-benefit pattern described above: training accrual maps more directly onto higher-order interference suppression than onto basic perceptual–motor speed.

This pattern is consistent with the motor-learning literature on skill-acquisition timescales. Perceptual–motor parameters such as basic reaction speed and movement smoothness typically reach near-ceiling within months of structured practice, whereas executive-control adaptations associated with prefrontal function appear to accrue more gradually, on the years-long timescale required for the consolidation of strategic, anticipation-based, and decision-related skills in racket sports. The fact that the strongest dose–response signal in the present dataset emerged on the conflict-effect index, rather than on baseline RT, is consistent with this distinction between fast-saturating low-level adaptations and slower-accruing higher-order ones. Whether further training years would continue to reduce the conflict effect linearly, or whether a ceiling emerges with extended training beyond the three-year window sampled here, cannot be determined from the present cross-sectional, restricted-range data; longitudinal studies covering both shorter (<1 year) and longer (>5 years) training durations are needed to characterize the full dose–response curve.

### 4.4 Comparison with prior findings

The magnitude of the present conflict-effect difference (*d* = 1.41 for RT; *d* = 0.91 for error rate) merits direct comparison with prior literature. A recent meta-analysis of cognitively engaging physical activity in children and adolescents (23 randomized trials, *N* = 2,857) reported a pooled effect on inhibitory control of SMD = 0.35 (Mao et al., 2024), and a meta-analysis of open-skill exercise in children and adolescents aged 5–16 (17 studies, *N* = 1,298) reported small-to-moderate effects on the same construct (Hu et al., 2024). Against these meta-analytic benchmarks, the between-group difference observed here is substantially larger.

Four factors plausibly contribute to this discrepancy. First, the meta-analyses synthesized intervention studies in which a typical participant accrued exposure on the order of weeks to a few months, whereas the present training-group participants had accumulated 1–3 years of cumulative training, roughly an order of magnitude longer. Second, the control contrast was deliberately stringent: instead of comparing two active conditions or pre- versus post-intervention scores, trained adolescents were compared against age-matched peers with negligible structured exercise, an extreme-group sampling strategy that necessarily widens the between-group gap relative to randomized trials in which both arms receive some baseline activity. Third, the 12–15-year window may amplify training-related plasticity relative to the younger or older samples typical of mixed-age intervention trials. Fourth, the cross-sectional design admits self-selection: adolescents with stronger baseline inhibitory control may be over-represented among those who initiate and persist with badminton training, which would inflate the observed contrast.

Taken together, the present effect sizes are best interpreted as an upper bound on the true causal influence of badminton training on adolescent inhibitory control, rather than as a typical or expectable training effect. The within-training-group dose–response correlation (*r* = −0.62 for training years against the RT conflict effect; *r* = −0.59 after excluding five influential observations, *n* = 27) provides an internally consistent, less self-selection-confounded estimate of the dose–cognition coupling, and converges in direction with the broader literature, while remaining correlational. Randomized controlled trials in this age range will be essential to triangulate the true causal effect against the cross-sectional upper bound reported here.

#### 4.4.1 Shape of the dose–response curve

A separate interpretive question concerns the shape of the dose–response relationship within the amateur training range. Three *a priori* possibilities can be entertained. The first is a monotonic linear curve, under which additional training years and additional weekly sessions would continue to reduce the conflict effect indefinitely. The second is a saturating curve, under which gains accrue rapidly during the first year or two and then plateau as the inhibitory system approaches an experience-dependent ceiling. The third is an inverted-U, under which moderate volumes optimize conflict-monitoring efficiency while very high volumes (typical of elite training) may produce no further gain or even modest dis-benefits attributable to cumulative fatigue or selection pressures unrelated to cognition.

The present cross-sectional dataset, restricted to 1–3 training years and three to five weekly sessions, cannot distinguish among these possibilities. The training-years correlation observed here (*r* = −0.62) is compatible with the linear and the not-yet-saturating segment of either of the latter two curves. Two features of the broader exercise-cognition literature nevertheless suggest a saturating shape may be the most plausible default. First, randomized-trial meta-analyses report that cognitively engaging interventions produce reliable but moderate gains within 6–12 weeks of training (Mao et al., 2024), implying a steep early ascent. Second, the open-skill meta-analysis in children and adolescents identifies three to five sessions per week as optimal (Hu et al., 2024), with diminishing or unstable returns at higher volumes. The present amateur sample, training at three to five sessions per week for 1–3 years, occupies the plausibly active portion of such a curve. Whether the conflict effect continues to shrink with five or more years of training, or whether it stabilizes at a level set during the first 1–2 years, is an empirical question that the present design cannot resolve. Longitudinal cohorts spanning the full range from sport initiation through late adolescence will be needed.

### 4.5 Limitations

Several limitations qualify the present conclusions. First, the cross-sectional between-group design cannot rule out self-selection bias: adolescents with stronger baseline inhibitory control may be more likely to initiate and persist with badminton training, which could inflate the observed between-group effect sizes; in addition, parental encouragement may jointly shape both badminton participation and executive development. Second, the present sample (*N* = 62) was drawn from a single city (Chengdu, China), which restricts generalizability to other regions, socioeconomic contexts, and ethnic backgrounds; replications in geographically diverse samples are needed before broader inferences can be drawn. Third, only behavioral measures were collected, so any discussion of prefrontal–cingulate mechanisms remains theoretical. Fourth, the unusually large effect size for the RT conflict effect (*d* = 1.41), although statistically robust given the >0.99 achieved power, exceeds typical magnitudes reported in the adolescent exercise–cognition literature (Best, 2010; Mao et al., 2024; Verburgh et al., 2014). It may reflect a combination of (a) heightened plasticity during this developmental window, (b) the marked contrast between trained adolescents and entirely non-exercising controls, (c) potential selection bias, and (d) the narrow extreme-group sampling strategy adopted here. Future longitudinal or randomized designs covering broader exposure ranges will be needed to disentangle these explanations. Fifth, the study did not collect or covary out lifestyle factors such as sleep quality, daily screen time, academic workload, daily non-sport physical activity, parental support, dietary patterns, parental education, or socioeconomic status, each of which can independently influence inhibitory control in adolescents. Sixth, training-group participants varied in coaching quality, training emphasis (e.g., technique drills versus match practice), and competition exposure, all of which are sources of within-group heterogeneity that the present design could not isolate. Seventh, handedness was determined by self-report rather than by a standardized inventory such as the Edinburgh Handedness Inventory. Eighth, the conflict-effect difference score showed only modest split-half reliability (0.38; the reliability paradox, Hedge et al., 2018), even though the underlying condition-level RTs were highly reliable (0.81–0.96); because low reliability attenuates correlations, the within-group dose–response estimate (*r* = −0.62) should be regarded as a conservative lower bound, and future individual-difference analyses should use more trials or hierarchical/latent-variable estimation.

An additional consideration concerns the demographic equivalence checks reported in §3.1. Although the standard null t tests on age, height, weight, and BMI returned *p* > 0.05, two one-sided tests of equivalence (TOST) at a SESOI of *d* = 0.5 returned *p* values in the 0.058–0.217 range; none reached the formal equivalence threshold. The demographic balance between groups should therefore be read as having failed to detect a difference at *α* = 0.05, rather than as having confirmed equivalence within ±0.5 *SD*. Larger samples would be needed to support a positive equivalence claim.

### 4.6 Practical implications and conclusion

The present data are consistent with, although they cannot causally establish, the proposition that amateur badminton training of at least three sessions per week, each lasting approximately 60 min, sustained over 1–2 years, may support the development of interference control in adolescents aged 12–15.

Because the present evidence is cross-sectional, it does not warrant curricular, programmatic, or family-level prescriptions. At most, the pattern is hypothesis-generating: the selectivity of the advantage, larger for the conflict effect than for basic processing speed, is consistent with Diamond and Ling’s (2016) argument that cognitively engaging activities yield the greatest executive benefits, and it motivates, without justifying, future cluster-randomized and longitudinal trials of open-skill racket training in adolescents.

Three priorities for future work follow directly. First, longitudinal or cluster-randomized designs are needed to address self-selection and to estimate the causal effect with appropriate precision. Second, the incorporation of neuroimaging or electrophysiological measures will help test the prefrontal–cingulate mechanism proposed here as a theoretical anchor (Botvinick et al., 2001). Third, dose–response sampling should extend beyond the 1–3-year window examined in the present amateur sample, to establish whether the conflict-effect benefit grows monotonically or saturates with extended training.

## Data Availability

The original contributions presented in the study are included in the article/supplementary material, further inquiries can be directed to the corresponding author/s.
